# Empowering women's health with miRNA-integrated nanochemical approaches: from reproductive health to cancer care

**DOI:** 10.1039/d5ra07723k

**Published:** 2026-04-21

**Authors:** Neda Farzizadeh, Zahra Najmi, Morteza Amoozgar, Mona Aminbeidokhti, Amirali Hariri, Arezoo Khosravi, Siavash Iravani, Ali Zarrabi

**Affiliations:** a Department of Midwifery, School of Nursing and Midwifery, Ardabil University of Medical Sciences Ardabil Iran; b Graduate School of Biomedical Sciences, Clinical and Translational Science Department, Tufts Medical Center Boston MA USA zahra.najmi@tuftsmedicine.org; c Associate Professor of Obstetrics and Gynecology, Fellowship in Minimally Invasive Surgery, Zanjan University of Medical Sciences Zanjan Iran; d CEO, March Health Company Concord CA 94521 USA; e Department of Pathology, University of California San Francisco San Francisco CA 94143 USA; f Department of Pharmaceutical Biotechnology, School of Pharmacy and Pharmaceutical Sciences, Isfahan University of Medical Sciences Isfahan 8174673461 Iran; g Department of Genetics and Bioengineering, Faculty of Engineering and Natural Sciences, Istanbul Okan University Istanbul 34959 Turkey; h Graduate School of Biotechnology and Bioengineering, Yuan Ze University Taoyuan 320315 Taiwan; i Independent Researcher W Nazar ST, Boostan Ave Isfahan Iran siavashira@gmail.com; j Department of Biomedical Engineering, Faculty of Engineering and Natural Sciences, Istinye University Istanbul 34396 Turkey alizarrabi@gmail.com ali.zarrabi@istinye.edu.tr

## Abstract

MicroRNAs (miRNAs), a category of small (18–25 nucleotides) non-coding transcripts that modulate gene expression at the post-transcriptional level, are necessary for regulatory processes in female reproduction, specifically in ovarian function and follicular development. Dysregulation of these miRNAs is implicated in reproductive disorders like PCOS, endometriosis, and gynecological cancers. Nanotechnology in medicine encompasses the study of the properties and uses of nanomaterials to enable the development of more precise therapeutic techniques, including biosensing and diagnostics, targeted medication delivery, and tissue engineering. In this review, the recent advancements in utilizing nano-based technologies accompanied by miRNAs, including polymeric nanostructures, metallic nanoparticles (NPs), extracellular vesicle (EV) mimetics, DNA-based nanostructures, and lipid-based nanostructures, to treat conditions like endometriosis and PCOS or breast, cervical, and ovarian cancers and their limitations are discussed. Despite the reported improvements, clinical translation is hampered by challenges related to long-term stability, scalability, and immune recognition. Future research should focus on improving hybrid nanocarrier systems and miRNA-based precision medicine to improve treatment outcomes for women's health.

## Abstract 2

This review summarizes the recent nano-based miRNA technologies for treating endometriosis, PCOS, and cancers, along with their current challenges and limitations.

## Graphical abstract

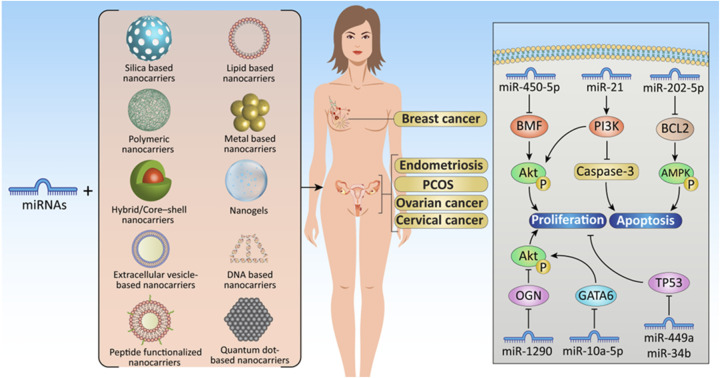

## 1. MicroRNAs in female reproductive biology

Women's health encompasses a broad spectrum of physiological processes, including hormonal regulation, reproductive function, pregnancy, metabolic balance, and aging.^1^ At the molecular level, these processes are tightly regulated by multiple pathways, among which microRNAs (miRNAs) play a pivotal role. miRNAs contribute to the maintenance of normal female physiology by modulating hormone signaling pathways, ovarian function, endometrial receptivity, and immune adaptations specific to women.^2–4^ Dysregulation of these miRNA-mediated pathways can therefore disrupt physiological homeostasis and contribute to the development of female-related diseases. Accordingly, understanding the role of miRNAs provides valuable insights not only into disease mechanisms but also into the preservation of women's health and the development of diagnostic and therapeutic strategies for female-specific disorders.

**Fig. 1:**
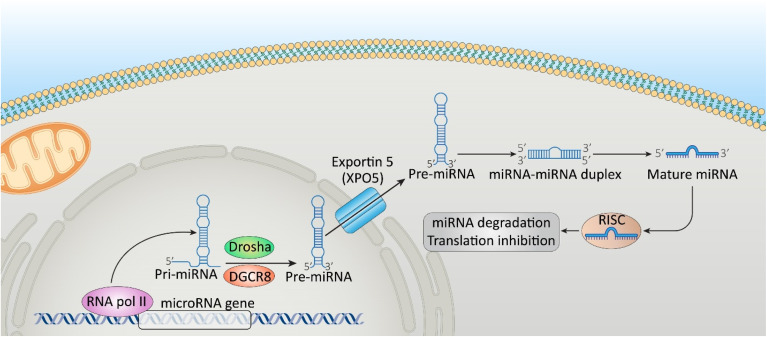
Biogenesis of microRNAs (miRNAs) and their role in the post-transcriptional level of gene expression.

miRNAs are a category of small (18–25 nucleotides) non-coding transcripts that modulate gene expression at the post-transcriptional level.^5^ The enzymes DiGeorge syndrome critical region 8 (DGCR8) and Drosha ribonuclease III (DROSHA) cleave primary miRNAs (pri-miRNAs) to produce precursor miRNAs (pre-miRNAs).^6,7^ Pre-miRNAs are transported to the cytoplasm by exportin 5 and then they are processed further to produce an miRNA duplex. The guide strand creates an RNA-induced silencing complex (RISC), accompanied by Argonaute proteins. miRNAs, through their interaction with the 3′-untranslated region (3′-UTR), promote post-transcriptional gene silencing by driving translational repression or messenger RNA (mRNA) degradation.^8,9^Fig. 1 demonstrates the biogenesis of miRNAs and their role in the post-transcriptional level of gene expression.

miRNAs are necessary for regulatory processes in female reproduction, specifically in ovarian function and follicular development; for example, miR-21 is expressed at high levels in mammalian ovaries and is critically associated with embryo development. It inhibits the apoptosis in cumulus and granulosa cells by activating the phosphatidylinositol 3-kinase/protein kinase B (PI3K/Akt) pathway and reducing the cleaved cysteine–aspartic acid protease 3 (caspase-3) levels.^10^ In mice, although miR-21 overexpression impairs cumulus expansion and reduces glutathione-*S*-transferase (GSH), it improves fertilization and blastocyst rates. Conversely, inhibiting miR-21 increases expansion and GSH but harms embryo development.^11^

**Fig. 2:**
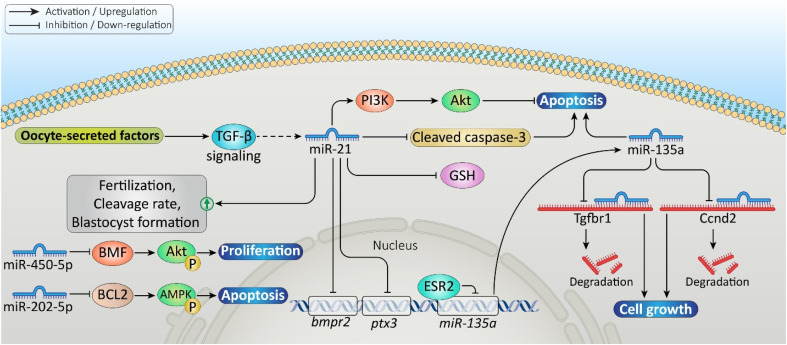
Role of the microRNAs (miRNAs) in ovarian function, follicular development, fertilization outcomes, and embryo development through the signaling pathways involved in cell proliferation or apoptosis.

Other miRNAs also regulate vital hormone receptors; for example, the expression of miR-34a negatively correlates with the follicle-stimulating hormone receptor (FSHR) during anestrus, and miR-let-7c inversely correlates with the estrogen receptor beta (ESR2) throughout the ovarian cycle.^12^ Moreover, miR-339-5p overexpression impairs oocyte maturation and blastocyst formation by reducing extracellular signal-regulated kinases 1 and 2 (ERK1/2) phosphorylation.^13^ In follicles, different expressions of miR-135a promote apoptosis and cell cycle arrest by targeting the transforming growth factor beta receptor 1 (TGFBR1) and cyclin D2 (CCND2). ESR2 directly suppresses the miR-135a transcription, revealing a pro-survival axis (ESR2/miR-135a/TGFBR1/CCND2).^14^ Furthermore, miR-20b and miR-31 target hydroxysteroid 17-beta dehydrogenase 14 (HSD17B14), altering steroid hormone conversion—estradiol to estrone—and impacting granulosa cell apoptosis. miR-20b reduces apoptosis, while miR-31 (which also targets FSHR) promotes it.^15^ In goats, miR-202-5p inhibits granulosa cell proliferation and promotes apoptosis, whereas miR-450-5p promotes proliferation by targeting B-cell lymphoma 2 (Bcl2) modifying factor (BMF) and Bcl2 genes, impacting Akt and adenosine monophosphate (AMP)-activated protein kinase (AMPK) signaling, respectively.^16^Fig. 2 demonstrates the role of miRNAs in ovarian function and follicular development.

Given their fundamental roles in folliculogenesis, oocyte maturation, steroidogenesis, and cell survival/apoptosis, dysregulation of these miRNAs is implicated in reproductive disorders, like polycystic ovary syndrome (PCOS), endometriosis, and gynecological cancers.^5,17–19^ These findings demonstrate the potential of miRNA-based therapeutic technologies in disease treatment and diagnosis. This has led to the development of synthetic miRNAs as potential therapeutic tools for restoring normal physiological levels of miRNA expression in a variety of illnesses. When low miRNA expression is the cause of a disease, miRNA mimics can bring miRNA levels up to normal, and antimiRNAs (antagomirs) can prevent disease-causing overexpressed miRNAs by preventing miRNAs from binding to their mRNA targets.^20–22^ To address these therapeutic methods, there is a need for nano-based technologies that can regulate miRNA levels that are dysregulated in diseases. In this review, we discuss the role of miRNAs in gynecological diseases and recent advancements in miRNA-based diagnostic and therapeutic pathways in nanotechnology.

### 1.1. Endometriosis

Endometriosis is a gynecological disease that is an inflammatory and estrogen-dependent condition defined by the presence of endometrial-like tissue outside the uterus, impacting between 5% and 10% of women of reproductive age, which can lead to pelvic discomfort and infertility. Women with this condition suffer from a wide variety of symptoms, including pelvic pain, dysmenorrhea, dyspareunia, heavy periods, fatigue, depression, and, in higher stages of endometriosis, adhesions, leading to infertility or ovarian endometriomas, which have a negative impact on ovarian reserve (oocyte count and quality) and the overall chance of successful conception. These symptoms influence the total quality of life of women.^23,24^ Understanding the role of miRNAs in both physiological and pathological processes in women's health may provide innovative solutions to manage conditions in a more specific and effective way to enhance the quality of life in women.

**Fig. 3:**
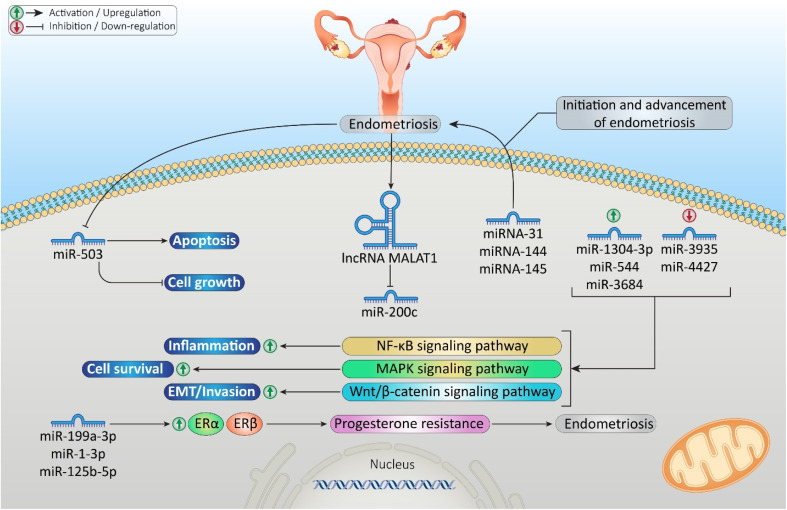
Role of microRNAs (miRNAs) in endometriosis initiation and advancement. The miRNAs were involved in the nuclear factor kappa-light-chain-enhancer of activated B cells (NF-κB), mitogen-activated protein kinase (MAPK), and wingless/integrated-beta-catenin (Wnt/β-catenin) pathways and progesterone resistance.

Different miRNAs have been reported to be expressed differentially in endometriosis; for example, apoptosis of endometriotic cyst stromal cells (ECSCs) is aided by miR-503.^25^ In addition, miR-200c was identified as significantly downregulated in ectopic endometrial tissues;^26^ in contrast, miR-199a-3p, miR-1-3p, miR-146a-5p, and miR-125b-5p were upregulated.^27^ Additionally, miRNAs such as miRNA-31, miRNA-144, and miRNA-145 have emerged as critical determinants in the initiation of endometriosis. Increased levels of miR-1304-3p, miR-544, and miR-3684 and decreased levels of miR-3935 and miR-4427 affected signaling pathways such as nuclear factor kappa-light-chain-enhancer of activated B cells (NF-κB), mitogen-activated protein kinase (MAPK), and wingless/integrated-beta-catenin (Wnt/β-catenin).^28^Fig. 3 demonstrates the role of miRNAs in the initiation and advancement of endometriosis.

### 1.2. Polycystic ovary syndrome

Polycystic ovary syndrome (PCOS) is an endocrine disorder in reproductive age that affects 4–20% of women globally.^29^ The pathophysiology of PCOS is not yet fully understood, but it seems that insulin plays a key role in its development.^30^ Women with this endocrine disorder experience improper periods, heavy periods, growth of hair, acne, darkening of the skin, headaches, and, in more progressed stages, infertility, metabolic syndrome, and cancer.^31^ Unrevealing the role of miRNAs in the maintenance of physiological status in the endocrinological system of women, besides the etiology of disease development through miRNA dysregulations, will provide novel areas in the management of the condition in a more patient-based approach with a focus on personalized medical plans.

Folliculogenesis, steroidogenesis, and cellular adhesion are important pathways that miRNA modulates and are believed to play a role in the pathophysiology of PCOS.^32^ For example, miR-223-3p was recognized as an essential regulator of insulin, inflammation, and autophagy pathways in PCOS.^33^ A positive correlation was observed between miRNA-125a-5p expression and low-density lipoprotein (LDL) levels in the PCOS group; miRNA-125a-5p was involved in regulating cholesterol biosynthesis, glycolysis, insulin receptor signaling, oxidative stress-induced senescence, and estrogen-dependent gene expression.^34^ Besides, miR-93 and miR-216a levels were inversely associated with dipeptidyl peptidase 4 (DPP4) serum concentrations, which play a role in blood sugar regulation. miR-320a levels were significantly lower in PCOS patients.^35^ Long non-coding RNA (lncRNA) of small nucleolar RNA host gene 12 (lncRNA SNHG12) overexpression was found to influence pathological alterations in PCOS rats using an insulin-treated KGN granulosa-like tumor cell model and a dehydroepiandrosterone (DHEA)-induced PCOS rat model. Furthermore, it suppressed the expression of miR-129 and miR-125b, while lncRNA SNHG12 had the opposite effect. In cell experiments, silencing the lncRNA SNHG12 decreased cell viability, inhibited proliferation, and increased apoptosis. Further analysis using a pull-down assay demonstrated that lncRNA SNHG12 interacted with miR-129 and miR-125b, confirming their binding sites.^36^

### 1.3. microRNAs in gynecological cancers

Gynecological cancers are cancers that develop in the female reproductive organs, including cancers of the ovaries, fallopian tubes, uterus/endometrium, cervix, vagina, and genitals, and each of them is accompanied by its own signs, symptoms, and risk factors. These types of cancers are a major public health issue and are highly prevalent among women of all ages. Unfortunately, patients are often diagnosed at an advanced stage, and this can be due to a variety of reasons, including a lack of awareness of or attention to symptoms.^37,38^ miRNAs offer hope for the development of novel, sensitive, and specific biomarkers that enable a better understanding of the molecular pathogenesis of gynecological cancers.^39^ To date, many detailed studies have been conducted to identify and characterize miRNAs, which are not only key diagnostic indicators but also prognostic and predictive.

#### 1.3.1. Ovarian cancer

**Fig. 4:**
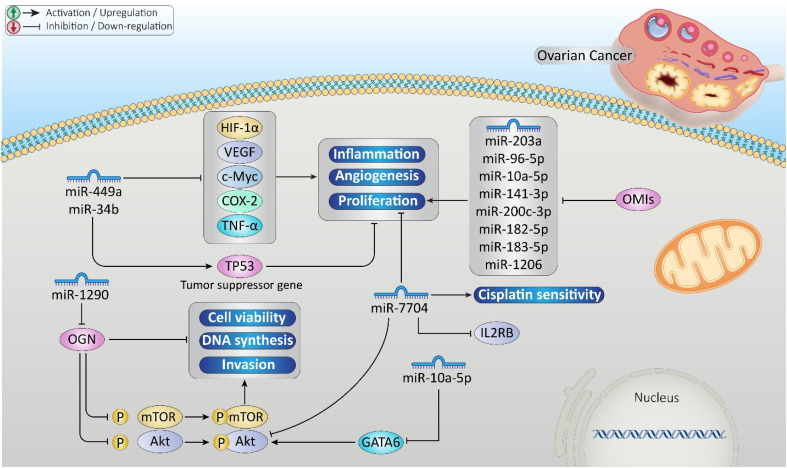
Role of the microRNAs (miRNAs) in ovarian cancer development and treatment response. The miRNAs were involved in inflammation, angiogenesis, proliferation, cisplatin sensitivity, and the protein kinase B/mechanistic target of rapamycin signaling (Akt/mTOR) pathway regulation.

Approximately 314 000 women globally receive an ovarian cancer diagnosis each year, resulting in 207 000 fatalities,^40^ and it remains the most lethal gynecological cancer.^41^ A wide variety of investigated dysregulated miRNAs in ovarian cancer; miRNAs like miR-141, miR-182, miR-200a, miR-20, miR-223, and miR-1246 were upregulated in ovarian cancer cells by promoting tumor growth, metastasis, and treatment resistance to cisplatin and paclitaxel.^42–48^ In contrast, miRNAs like the let-7 family were downregulated by tumor suppression through downregulation of Kirsten rat sarcoma viral oncogene homolog (KRAS) and cellular myelocytomatosis oncogene (c-Myc).^49^ Overexpression of miR-449a and miR-34b inhibited cancer-associated processes by upregulating the tumor suppressor gene TP53 and downregulating oncogenes, such as hypoxia-inducible factor 1-alpha (HIF-1α), vascular endothelial growth factor (VEGF), c-Myc, cyclooxygenase-2 (COX-2), and tumor necrosis factor-alpha (TNF-α).^50^ Additionally, miR-200a-3p, miR-1246, miR-203a-3p, and miR-23b-3p had higher expression levels nearer the diagnosis, with miR-200a-3p exhibiting statistically significant upregulation.^51^ miR-203a, miR-96-5p, miR-10a-5p, miR-141-3p, miR-200c-3p, miR-182-5p, miR-183-5p, and miR-1206 were consistently expressed in both high-grade serous ovarian cancer (HGSOC) cell lines and formalin-fixed paraffin-embedded human ovarian tumor samples.^52^ It is reported that overexpression of osteoglycin (OGN) in cancer-associated fibroblasts (CAFs) reduced ovarian cancer cell viability. One miRNA that has been found to target and inhibit OGN expression is miR-1290.^53^ Besides, upregulation of miR-10a-5p inhibited ovarian cancer cell proliferation by reducing GATA-binding protein 6 (GATA6) expression.^54^ Additionally, it is found that GATA6 was negatively correlated with miRNA-10a-5p in ovarian cancer tissues and cells.^54^ Moreover, miR-7704 was significantly downregulated in cisplatin-resistant ovarian cancer cells compared to parental cells. The ectopic expression of miR-7704 produced effects similar to interleukin-2 receptor subunit beta (IL2RB) knockdown.^55^ Mechanistically, miR-7704 inhibited ovarian cancer progression by regulating IL2RB expression.^56^Fig. 4 demonstrates the role of miRNAs in ovarian cancer development and treatment response.

#### 1.3.2. Cervical cancer

Approximately 660 000 new cases of cervical cancer were reported in 2022, making it the fourth most common cancer diagnosed in women worldwide, leading to serious health challenges.^57,58^ The mechanisms of miRNA dysregulation and their expression patterns in cervical cancer have been clarified by a number of studies; for example, miR-124-3p, miR-143, miR-145, miR-424, miR-491-5p, miR-494, miR-494, miR-497, and miR-544 suppressed tumors by increasing cell cycle arrest and decreasing proliferation.^7,59–63^ Additionally, miR-1246, miR-221, miR-93-5p, miR-10a, and miR-17-5p stimulate tumors to grow.^64–68^ Furthermore, the expression of lncRNA H19 increased in cervical cancer cells. miR-140, interacting with lncRNA H19, which is primarily found in the nucleus, was downregulated in cervical cancer cells. The effects of lncRNA H19 knockdown on cervical cancer cell development were reversed by inhibiting miR-140. miR-140 inhibited aldehyde dehydrogenase 1 family member A1 (ALDH1A1), and lncRNA H19 knockdown decreased ALDH1A1 expression.^69^ Moreover, it was found that miR-145 negatively correlated with metabolic reprogramming-related genes and inhibited the proliferation and metastasis of cervical cancer cell lines by disrupting aerobic glycolysis, a hallmark of cancer cell metabolism. miR-145 directly binds to the 3′-UTR of the myelocytomatosis oncogene (Myc). Additionally, Myc regulates glycolysis-related genes. *In vivo* experiments revealed that miR-145 mimics significantly suppressed cervical cancer xenograft tumor growth, prolonged the survival time of mice, and reduced the expression of the tumor proliferation marker Ki-67.^70^ It has been reported that overexpressing miRNA-143 enhances cisplatin-induced apoptosis linked to the modulation of apoptosis-related genes, such as Bcl2, Bcl2-associated X protein (Bax), and caspase-9, induces cell cycle arrest at the G1 and G2/M phases, activates autophagy, and inhibits cell migration by downregulating vimentin and downregulating c-Myc oncogene.^71^

#### 1.3.3. Breast cancer

Around 670 000 female breast cancer deaths and 2.3 million new cases were reported worldwide in 2022.^72^ Deep sequencing and profiling of miRNAs have shown significant miRNA dysregulation in breast cancer in recent years.^73^ For example, it is found that miR-25-3p, miR-29a-5p, miR-105-3p, and miR-181b1-5p were upregulated whereas miR-335-5p and miR-339-5p were downregulated in tumor tissues. The expression of these miRNAs was associated with Tumor–Node–Metastasis (TNM) stages and Human Epidermal Growth Factor Receptor 2 (HER-2) status, except for miR-105-3p and miR-339-5p.^74^ Both miR-450a-5p and miR-181a-3p were upregulated in the serum of breast cancer patients. In contrast, miR-450a-5p also showed elevated expression but did not display the same level of clinical relevance.^75^ It is found that miR-21, miR-155, miR-23a, miR-130a, miR-145, miR-425-5p, and miR-139-5p were significantly upregulated, while miR-451 was downregulated in patients with early invasive ductal carcinoma (IDC) patients.^76^ Targeting acyl-CoA synthetase long-chain family member 4 (ACSL4), which contributes to doxorubicin (DOX) resistance by controlling the drug extrusion pump adenosine triphosphate (ATP)-binding cassette subfamily G member 2 (ABCG2), miR-449 dysregulates fatty acid metabolism.^77^ Inhibiting miR-1307-3p significantly reduces cell proliferation, migration, invasion, and angiogenesis by regulating protamine 2 (PRM2) expression.^78^

Table 1 summarizes miRNAs and their regulation in endometriosis, PCOS, and gynecological cancers (ovarian, cervical, and breast cancers).

**Table 1:** Summary of miRNAs and their regulation in endometriosis, PCOS, and gynecological cancers (ovarian, cervical, and breast cancers)

	miRNA	Sample type	Up-/down-regulation	Ref.
Endometriosis	miR-503	Endometrial tissue	Downregulated	26
	miR-200c	Endometrial tissue	Downregulated	26
	miR-144	Endometrial tissue	Upregulated	28
	miR-145	Endometrial tissue	Upregulated	28
	miR-1304-3p	Endometrial tissue	Upregulated	28
	miR-544	Endometrial tissue	Upregulated	28
	miR-3684	Endometrial tissue	Upregulated	28
	miR-3935	Endometrial tissue	Downregulated	28
	miR-4427	Endometrial tissue	Downregulated	28
	miR-199a-3p	Endometrial tissue	Upregulated	27
	miR-1-3p	Endometrial tissue	Upregulated	27
	miR-146a-5p	Endometrial tissue	Upregulated	27
	miR-125b-5p	Endometrial tissue	Upregulated	27
PCOS	miR-223-3p	Granulosa cells samples	Upregulated	33, 79 and 80
	miR-93	Serum	Downregulated	35
	miR-216a	Serum	Downregulated	35
	miR-320a	Serum	Downregulated	35
Ovarian cancer	miR-141	Ovarian cancer cell	Upregulated	42–48
	miR-182	Ovarian surface, fallopian tube secretory cells, and malignant ovarian cell lines	Upregulated	42–48
	miR-200a	Ovarian adenocarcinoma cell lines	Upregulated	42–48
	miR-223	Tumor-associated macrophages	Upregulated	42–48
	miR-1246	Ovarian cancer cells	Upregulated	42–48
	Let-7 family	Ovarian cancer cells	Downregulated	49 and 81
	miR-449a	Endometrial and ovarian cancer cells	Downregulated	50
	miR-34b	Endometrial and ovarian cancer cells	Downregulated	50
	miR-203a-3p	Serum	Upregulated	51
	miR-23b-3p	Serum	Upregulated	51
	miR-1290	Cancer-associated fibroblasts	Upregulated	53
	miR-10a-5p	Ovarian tumor samples	Upregulated	52
	miR-7704	Ovarian cancer cells	Downregulated	56
Cervical cancer	miR-124-3p	Cervical cancer cells	Downregulated	7 and 59–63
	miR-143	Cervical cancer cells	Downregulated	7 and 59–63
	miR-145	Cervical cancer cells	Downregulated	7 and 59–63
	miR-424	Cervical cancer cells	Downregulated	7 and 59–63
	miR-491-5p	Cervical cancer cells	Downregulated	7 and 59–63
	miR-494	Cervical cancer cells	Downregulated	7 and 59–63
	miR-497	Cervical cancer cells	Downregulated	7 and 59–63
	miR-544	Cervical cancer cells	Downregulated	7 and 59–63
	miR-1246	Cervical cancer cells	Upregulated	64–68
	miR-221	Blood and cervical cancer cells	Upregulated	64–68 and 82
	miR-93-5p	Cervical cancer cells	Upregulated	64–68
	miR-10a	Cervical cancer cells	Upregulated	64–68
	miR-17-5p	Cervical cancer cells	Upregulated	64–68
	miR-140	Cervical cancer cells	Downregulated	69
	miR-141-5p	Cervical cancer cells	Upregulated	83
	miR-34a	Cervical cancer cells	Downregulated	84
Breast cancer	miR-25-3p	Breast cancer cells	Upregulated	74 and 85
	miR-29a-5p	Breast cancer cells	Upregulated	74
	miR-105-3p	Breast cancer cells	Upregulated	74
	miR-181b1-5p	Breast cancer cells	Upregulated	74
	miR-335-5p	Breast cancer cells	Downregulated	74
	miR-339-5p	Breast cancer cells	Downregulated	74
	miR-574-5p	Breast cancer cells	Upregulated	86
	miR-450a-5p	Serum	Upregulated	75
	miR-181a-3p	Serum	Upregulated	75
	miR-21	Breast cancer cells	Upregulated	76
	miR-155	Breast cancer cells	Upregulated	76
	miR-23a	Breast cancer cells	Upregulated	76
	miR-130a	Breast cancer cells	Upregulated	76
	miR-425-5p	Breast cancer cells	Upregulated	76
	miR-139-5p	Breast cancer cells	Upregulated	76
	miR-451	Breast cancer cells	Downregulated	76
	miR-449a	Breast cancer cell lines	Downregulated	77
	miR-449b-5p	Breast cancer cell lines	Downregulated	77
	miR-449c-5p	Breast cancer cell lines	Downregulated	77
	miR-1307-3p	Breast cancer cell lines	Upregulated	78

## 2. Nanotechnology in women's health

Nanotechnology in medicine encompasses the study of the properties and uses of materials at the nanometer scale (0.1–100 nm)^87^ to enable the development of more precise therapeutic techniques, including biosensing and diagnostics, targeted medication delivery, and tissue engineering.^87–89^ Nanoparticles (NPs) have been utilized to effectively transport miRNA-based therapeutics into cells or various cancer model organisms, either independently or in combination with chemotherapeutic agents, to attain a synergistic impact specifically in cancer treatment, minimizing their impact on normal cells.^90–93^ Prominent nanocarriers utilized in miRNA-based cancer therapies include liposomes, exosomes, dendrimers, mesoporous silica (MSN) NPs, quantum dots, gold nanoparticles (AuNPs), iron oxide nanoparticles (IONPs), and core–shell nanomaterials, among others.^94–105^ Furthermore, the authorization of nanotechnology platforms for the distribution of anti-cancer pharmaceuticals in the market, including Doxil, Caelyx, and Myocet (for DOX), DaunoXome (for daunorubicin), Mepact (for mifamurtide), and NanoTherm (for Fe_2_O_3_), has been granted.^106^ Recently, nanotechnology has gained attention in the treatment of gynecological cancers. In the following sections, recent advancements in this field are discussed.

### 2.1. Nanocarrier-based microRNA delivery in gynecological cancer treatment

#### 2.1.1. Breast cancer

Polyethylene glycol (PEG) has constituted the primary components of various liposomal formulations owing to its biocompatibility, solubility, and stability^107^ and is utilized to enhance the circulation duration of liposomes, which can be integrated into the liposome surface using a cross-linking lipid;^108^ for example, Ahmadi *et al.* recently developed a nano-delivery system using Span 60, cholesterol, a cationic lipid (CTAB), and DSPE-PEG2000 encapsulating AuNPs and miRNA-33a mimic. The miRNA mimic sequences were synthesized by Takapouzist (Tehran, Iran). In this study, it was shown that the components of the cationic niosome formulation mechanically tuned the physicochemical properties of the nanocarrier. CTAB significantly reduced the size of the vesicles by creating electrostatic repulsion and increasing the curvature of the bilayer. Cholesterol showed a concentration-dependent effect; at high concentrations, it increased the vesicle size and thermodynamic stability by increasing the order of the lipid chains and reducing membrane fluidity. Additionally, the interaction between Span 60, cholesterol and CTAB modulated the size and zeta potential of the NPs by changing the surface free energy and membrane stiffness. It showed a polydispersity index (PDI) of 0.26–0.28 and a high encapsulation efficiency of 82.41%. A controlled two-phase drug release occurred, fitting well with the Higuchi kinetic model (*R*^2^ = 0.9996). It showed apoptosis of up to 73% through the Bax/Bcl2 pathways. The synergistic interaction between AuNPs and miRNA-33a confirmed a combination index (CI) lower than 1. It also had good colloidal stability over 90 days with minimal changes in size, zeta potential, and PDI.^109^ However, it should be considered that the lack of targeting ligands means that tumor targeting relies solely on the enhanced permeability and retention (EPR) effect, which may not be effective in all tumor types. This study was limited to *in vitro* assessments of MCF-7 cells, with no *in vivo* validation, which limited the immediate translation to clinical applications. The cytotoxicity assessment was limited to cancerous cells; there were no data on normal cell toxicity, so the potential off-target effects remained unclear.

Farhana *et al.* synthesized AuNPs by using the trisodium citrate method. These NPs were introduced into MCF-7 cells and were non-toxic up to a concentration of 0.6 µg mL^−1^. The main therapeutic mechanism was that AuNPs significantly increased the expression of hsa-miR-204-5p, which bound to the 3′-UTR regions of matrix metalloproteinase-9 (MMP-9) mRNA, inhibiting MMP-9 mRNA expression and protein secretion. This pathway was also associated with the suppression of the phorbol 12-myristate 13-acetate (PMA)-induced inflammatory activation of the transcription factor NF-κB p65. However, it should be noted that only MCF-7 breast cancer cells were used, and no animal models or clinical samples were examined to confirm their therapeutic effect *in vivo*. The evaluations were conducted for 72 hours, so the long-term effects and potential toxicity of the AuNPs remained unknown. In addition, a PDI value of 0.435 indicated a non-uniform size distribution of the NPs. Although AuNPs were reported to be non-toxic at low doses, systemic toxicity and off-target effects were not investigated. Additionally, the results were limited to estrogen receptor-positive breast cancer cells, and the effects on other breast cancer subtypes were not determined.^110^

Chaudhari *et al.* designed a PEG-capped AuNP system synthesized with diethylpyrocarbonate (DEPC) for miRNA-206 delivery to target Luminal-A type breast cancer cells. In this study, it was shown that PEGylated AuNPs played an active role in the biological function of miR-206. The attachment of NH_2_-PEG-SH to the AuNP surface increased the hydrodynamic size and changed the zeta potential from negative to positive, which allowed for the electrostatic binding of miR-206 through the interaction of the amino groups of PEG with the phosphate groups of miRNAs. The stable formation of the miRNA-PEG-AuNP nanocomplex was confirmed by a further increase in size and change in zeta potential, and the miRNA loading efficiency increased in a time-dependent manner and reached saturation after 24 hours. It showed a binding efficiency plateau (BEP) of around 76% at 24 hours and significant cytotoxicity in MCF-7 breast cancer cells, reducing cell viability by over 80% after 48 hours in a dose-dependent manner by inducing G0/G1 phase arrest, S and G2/M phase reduction and activating apoptosis pathways through upregulation of pro-apoptotic Bax, downregulation of anti-apoptotic Bcl2, and suppression of NOTCH. Functional studies showed that these effects were exerted through the activation of the mitochondrial-dependent apoptosis pathway, as evidenced by decreased mitochondrial membrane potential, increased Annexin V-positive cells, increased Bax expression, and decreased Bcl2 expression. However, it should be noted that the study did not assess caspase-6 and -7, leaving the exact apoptosis mechanism partially unresolved, and that the therapeutic efficacy and biosafety were only tested in MCF-7 cells, without *in vivo* validation or testing in other cancer cell lines. Additionally, the present BEP may be insufficient for certain therapeutic applications. Moreover, the lack of pharmacokinetic or biodistribution data to assess how the system behaves in systemic circulation, uptake in other tissues, or clearance was another limitation of the study.^111^

**Fig. 5:**
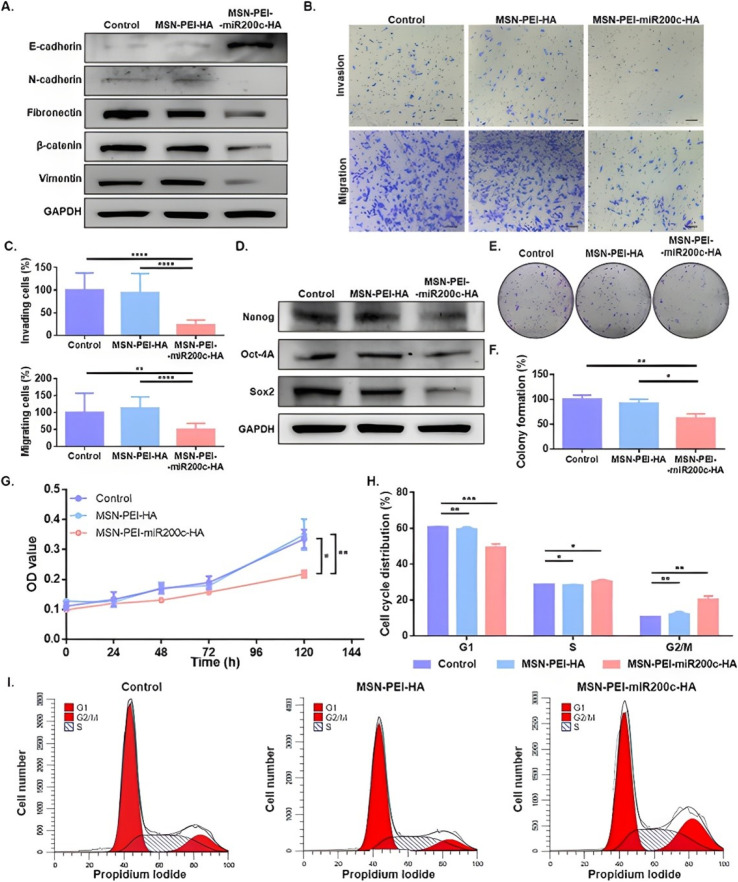
Effects of the MSN-PEI-miR200c-HA on EMT, invasion, migration, stem-like properties, colony formation, and the cell cycle. The MDA-MB-231 cells were treated with MSN-PEI-HA or MSN-PEI-miR-200c-HA for 72 h (20 µg mL^−1^) and untreated cells (control) were also included. (A) Protein expression levels of E-cadherin, N-cadherin, fibronectin, β-catenin, vimentin, and GAPDH. (B and C) Invasion and migration assays: representative images (scale bar = 100 µm) (B) and quantification profiles (mean ± SD) (C). (D) Protein expression levels of Nanog, Oct-4A, Sox2, and GAPDH. (E and F). Colony formation assays: representative images (E) and quantification profiles (mean ± SD) (F). (G) Cell proliferation analysis (mean ± SD). (H and I) Cell cycle analysis by flow cytometry: quantifications (mean ± SD) (H) and representative cell cycle profiles (I). OD: optical density and *p < 0.05; **p < 0.01; ***p < 0.001; and ****p < 0.0001. Reprinted with permission from ref. 112 ACS Applied Materials & Interfaces, licensed under CC BY 4.0.

**Fig. 6:**
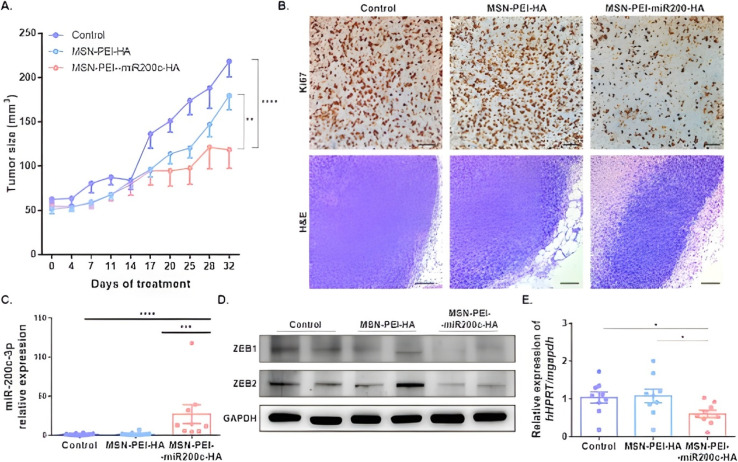
Antitumor activity of MSN-PEI-miR200c-HA *in vivo*. (A) Tumor growth rate in mice treated with PBS, MSN-PEI-HA, and MSN-PEImiR200c-HA (mean ± SEM, *N* = 27). (B) Representative images of the Ki-67 (scale bar = 50 µm) and H&E (scale bar = 100 µm) staining of the tumor sections. (C) miR-200c-3p expression in xenograft tumors determined by qRT-PCR (mean ± SEM). (D) Protein expression levels of ZEB1, ZEB2, and GAPDH in xenograft tumors. (E) hHPRT expression relative to mGAPDH in lung tissue determined by qRT-PCR (mean ± SEM). *p < 0.05; **p < 0.01; ***p < 0.001; and ****p < 0.0001. Reprinted with permission from ref. 112 ACS Applied Materials & Interfaces, licensed under CC BY 4.0.

An MSN/NP-based nanodevice was developed for the targeted delivery of miR-200c-3p. miR-200c-3p was acquired from Guangzhou RiboBio CO (Guangzhou, China). The final nanodevice (MSN-PEI-miR200c-HA) was prepared using a layer-by-layer procedure on the core of the MSN. A major challenge for miRNA carriers is being degraded in endosomes or lysosomes. To overcome this, polyethylenimine (PEI) was included; PEI becomes protonated when exposed to acidic pH inside endosomes or lysosomes, and it induces a proton sponge effect, which drives the NPs to escape from the endosomes or lysosomes into the cytoplasm. This ensures that the payload is delivered functionally. It showed an efficient miRNA loading with 99% binding and an efficient endosomal escape. *In vitro* studies confirmed this by showing the rapid release of a labeled miRNA mimic in lysosomal extract and, subsequently, the dispersion of the miRNA throughout the cytosol after 45 minutes of incubation with cells. As an active targeting mechanism, the outer hyaluronic acid (HA) coating targets the CD44 receptor, which is highly overexpressed in breast cancer cells and is involved in tumor progression. *In vitro* tests showed significantly higher uptake in CD44-high MDA-MB-231 cells compared to CD44-negligible OE19 cells. Delivery of miR-200c-3p resulted in induction of cell cycle arrest in the G2 phase and significant reduction in tumor size and metastasis *in vivo* with no observable toxicity confirmed by renal, hepatic, and body weight assessments. Upregulation of miR-200c-3p and downregulation of zinc finger E-box-binding homeobox 1 (ZEB1) and ZEB2 (master regulators of the epithelial to mesenchymal transition [EMT] involved in tumor progression, drug resistance, and metastasis) were confirmed in tumors, which shows the efficient delivery of functional miR-200c-3p *in vivo*. In biological fluids, NPs showed partial aggregation around 400 nm in hydrodynamic size after 1 hour. Despite effective tumor targeting, NPs accumulated not only in tumors but also in the lungs, spleen, liver, and kidneys, which could affect long-term safety. The miRNA content in the final NPs was 0.7 wt%, which can limit the dosing capacity. Although PEI facilitated endosomal escape, it was associated with dose-dependent cytotoxicity in many systems. NPs were stable in water but showed size increase in the culture medium, indicating that their physiological stability could be compromised and might need further formulation tuning. However, the study focused on xenografts that result in other cancer types, or patient-derived xenografts remain untested (Fig. 5 and 6).^112^

Bose *et al.* investigated the use of urokinase-type plasminogen activator (uPA) peptide-functionalized extracellular vesicles (EVs) to enhance the tumor-targeting ability of polymeric nanocarriers (PNCs) loaded with antimiRNA-21 or antimiRNA-10b. Cy5-labeled anti-miR-21, anti-miR-21, and anti-miR-10b RNA oligonucleotides were obtained from the PAN Facility at Stanford University. Antisense miRNA-21 and antimiRNA-10b were encapsulated within biodegradable poly(lactic-*co*-glycolic acid) (PLGA) NPs to protect them from degradation and enable prolonged intracellular availability, thereby restoring tumor suppressor signaling and suppressing invasion- and metastasis-associated pathways. Surface functionalization with EVs derived from 4T1 triple-negative breast cancer (TNBC) cells preserved native cancer cell adhesion molecules that mediated homologous tumor recognition and preferential uptake by TNBC cells. The EV membrane coating also reduced the burst release of antisense miRNAs from the PLGA core, which led to sustained gene silencing. When combined with low-dose DOX, which transiently slowed cell-cycle progression, this platform achieved synergistic antiproliferative effects by extending the functional window of miRNA-mediated pathway inhibition. Encapsulation efficiencies of antimiRNA-21 and antimiRNA-10b were 70% ± 5% and remained consistent across batches (65–75%). The plasminogen activator (PA) nanococktail combined with low-dose DOX produced a 2.8-fold higher antiproliferative effect compared with DOX alone and a 3.2-fold increase relative to untreated controls (*P* < 0.01). At the end of the study, 40% (4/10) of the mice exhibited complete tumor elimination. Notable accumulation of antimiRNAs in the liver, spleen, and kidneys suggested incomplete tumor specificity; therefore, long-term safety and immunogenicity were not assessed. Co-loading of multiple antimiRNAs and DOX presented challenges in achieving synchronized, controlled release although partial success was achieved.^113^

Schilb *et al.* created integrin-targeted, PEGylated lipid NPs composed of an Arg-Gly-Asp (RGD) peptide for receptor-mediated cellular uptake, PEG for steric stabilization and prolonged circulation, and a pH-responsive ionizable (1-aminoethyl)iminobis[*N*-oleicylcysteinyl-(aminoethyl)propionate] (ECO) lipid that enables electrostatic nucleic acid complexation and efficient intracellular release. Human miR-200c duplex and negative control small interfering RNA (siRNA) duplex were purchased from Integrated DNA Technologies (IDT, Coralville, IA). Self-assembly of RGD-PEG-MAL, the amphiphilic lipid ECO, and an immobilized miR-200c bistrand (nitrogen-to-phosphate ratio [N/P] = 8) resulted in uniformly sized, positively charged NPs with high miRNA encapsulation efficiency. RGD modification significantly enhanced cellular internalization and led to efficient cytosolic translocation, as demonstrated by confocal microscopy and a >360-fold increase in intracellular fluorescence within 4 hours. Stable transfection resulted in a prolonged elevation of miR-200c levels for up to 14 days, enabling effective suppression of targets associated with EMT, including ZEB1, B lymphoma mo-mlv insertion region 1 homolog (BMI1), survivin, and fibronectin, as well as the oncofetal extracellular matrix isoform extradomain B fibronectin (EDB-FN). miR-200c replacement reduced the migration, invasion, and 3D spheroid growth of TNBC cells. In orthotopic TNBC xenograft models, weekly systemic administration of RGD-PEG-ECO/miR-200c NPs significantly inhibited tumor progression without detectable toxicity, while molecular magnetic resonance (MR) imaging with the EDB-FN-targeted contrast agent, ZD2-N3-Gd(HP-DO3A) (MT218), confirmed treatment-associated remodeling of the tumor microenvironment. Within 24 hours, miR-200c levels in MDA-MB-231 cells rose almost 1000 times, remained at high expression for 4 days, dropped to 80 times by day 7, and stayed 30 times higher than controls for 14 days. Additionally, Hs578T cells showed a notable upregulation, with increases of 50–100 times at 48 hours. The expression of ZEB1 mRNA decreased by 40% in Hs578T cells and 57% in MDA-MB-231 cells. Survivin levels fell by 25% and 64%, while BMI1 expression fell by 15% and 35%, respectively. In contrast to the around 3-fold increase in control-treated tumors, tumor volumes in MDA-MB-231 xenografts stayed relatively stable (76.1 ± 15.2 mm^3^ to 111.4 ± 22.1 mm^3^, *P* > 0.05). In Hs578T xenografts, treated tumors showed markedly slower progression, whereas control tumors grew 2.2 times. Besides the positive results, the study had a number of limitations. The first was the temporary character of miR-200c expression, which dropped precipitously by day 7 and only moderately by day 14, requiring weekly systemic administration to maintain therapeutic effects. Another limitation was the variation in the response pattern of TNBC cell lines; the treatment of highly aggressive TNBC subtypes may be limited considering that Hs578T cells are more aggressive, which showed relatively lower suppression of EDB-FN and smaller tumor volume reductions. *In vivo*, total tumor regression was not achieved, particularly in Hs578T models, where treatment-induced residual EDB-FN expression persisted. Additionally, one mouse in the treatment group unexpectedly died despite the absence of any obvious toxic effects, underscoring the need for more comprehensive safety assessments. The use of preclinical murine models, which might not fully capture the biology of human TNBC, presents another difficulty.^114^

Albakr *et al.* created a nanoliposome (NL) with miR-1296 (NL-miR-1296). Cationic nanoliposomes containing 1,2-dioleoyl-3-trimethylammonium-propane (DOTAP) were designed to deliver miR-1296 *via* electrostatic interaction and provided high encapsulation efficiency and formation of unilamellar vesicles with a size of 120–135 nm, providing stability and effective cellular uptake. The miR-1296 mimic (accession no. MIMAT0005794) and control miRNA (NC) were purchased from Life Technologies (Carlsbad, California, United States), and fluorescently labeled miR-1296-CY3 conjugate was purchased from Invitrogen (Carlsbad, California, United States). An N/P ratio of 3 protected miRNA against serum degradation and controlled release (around 33% over 24 hours). Encapsulation efficiency was 94.47% in N/P by 3. NL-miR-1296 entered the cells in a time-dependent manner and was released into the cytoplasm, where it downregulated cyclin D1 (CCND1) and poly(ADP-ribose) polymerase 1 (PARP1) expression and reduced TNBC cell viability. Furthermore, pretreatment with NL-miR-1296 significantly increased the sensitivity of the cells to cisplatin. In MDA-MB-231 cells, 0.24 µM of NL-miR-1296 significantly reduced viability *versus* untreated cells (*P* < 0.001), 0.5 µM of NL-miR-1296 reduced viability to 33.45% ± 5.29% *versus* 94.94% ± 14.68% with NL-miR-NC (*P* < 0.002), and lipofectamine did not significantly reduce viability at comparable doses. Additionally, 0.5 µM NL-miR-1296 combined with 1 µM cisplatin reduced viability significantly (*P* < 0.001). Up to 40 days, no significant loss of biological activity was reported, but reduced activity was observed after 80 days at 25 °C (*P* < 0.002) and 4 °C (*P* < 0.05). It should be noted that serum protection was evaluated for only up to 24 hours. Moreover, the study was limited to *in vitro* experiments on only the MDA-MB-231 TNBC cell line, with no animal model data on therapeutic efficacy or pharmacokinetics.^115^

A single-stranded DNA template was designed containing antisense sequences for miR-182 and miR-205. They synthesized manganese dioxide (MnO_2_) NPs by reducing potassium permanganate (KMnO_4_) using a PAH (cationic polyelectrolyte). They then attached Ce6 (chlorin e6), creating MnO_2_@Ce6 NPs. To provide a hydrogel loaded with doxorubicin (HD), they loaded DOX into the hydrogel by incubating the hydrogel with DOX. To provide HDMnO2@Ce6 (HDMC), they bound MnO_2_@Ce6 particles electrostatically to the hydrogel. The highest killing effect was observed in HDMC combined with light (660 nm light [50 mW cm^−2^], 10 minutes), followed by HDMC alone, and the lowest was reported for hydrogel. Some of the limitations of this innovation were the unstable nature of MnO_2_@Ce6 in biological media, leading to coagulation; however, the hydrogel component stabilized MnO_2_@Ce6. This system could still face stability challenges in complex biological environments. Besides, this system targeted MDA-MB-231 and its potential off-target effects in other cancer types or heterogeneous tumors are not extensively discussed. The follow-up duration was 14 days in murine models, lacking long-term toxicity data. In addition, the multi-step synthesis process could present scalability challenges for clinical translation. It should be noted that the use of cytosine–phosphate–guanine (CpG) sequences may induce immune responses, but immune-related outcomes were not assessed. The photodynamic therapy (PDT) effect relies on a 660 nm laser, which limits tissue penetration (about 5–10 mm), posing a challenge for treating deep tumors.^116^

**Fig. 7:**
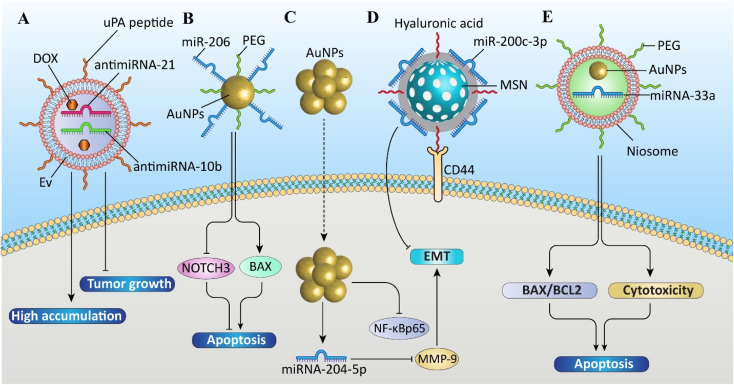
Schematic of the various nanoparticle (NP)-based delivery systems for microRNA (miRNA) therapeutics in breast cancer. From left to right: (A) extracellular vesicle (EV)-based nanocarrier co-delivering doxorubicin (DOX), antimiRNA-21, and antimiRNA-10b, functionalized with urokinase-type plasminogen activator (uPA) peptide; (B) gold nanoparticles (AuNPs) conjugated with polyethylene glycol (PEG) and miR-206; (C) AuNPs delivering miRNA-204-5p; (D) mesoporous silica nanoparticles (MSNs) coated with hyaluronic acid and loaded with miR-200c-3p; and (E) niosome-based PEGylated delivery of miR-33a and AuNPs.

Fig. 7 demonstrates the schematic of various NP-based delivery systems for miRNA therapeutics in breast cancer. Moreover, Table 2 summarizes the NP-based miRNA delivery systems in breast cancer research.

**Table 2:** Summary of the nanoparticle-based miRNA delivery systems in breast cancer research

System name	Samples	Key data (size, zeta potential, and EE%)	Main statistical data (viability, toxicity, and efficacy)	Challenges/limitations	Year (ref.)
HDMC (hydrogel + DOX + MnO_2_@Ce6)	MDA-MB-231, L02, MCF-7, and 4T1 (*in vivo*)	Size: HDMC 190 nm; zeta: ∼−17.8 mV; and DOX EE: 100 µM saturation	HDMC + light → highest cytotoxicity and *in vivo*: slowest tumor growth, reduced metastasis, and minimal weight loss	Instability in biological media, limited tumor model, short-term (14 days) study, and complex synthesis	2021 (ref. 116)
NL-miR-1296 (niosomal lipoplex)	MDA-MB-231	Size: 123–135 nm; zeta: positive (DOTAP); and EE: 94.47% (N/P 3)	0.5 µM NL-miR-1296 → 33.45% viability; sensitized to cisplatin; and high uptake	*In vitro* only, 24 h serum stability, instability after 80 days storage, and no *in vivo* data	2021 (ref. 115)
RGD-PEG-ECO/miR-200c nanoparticles	MDA-MB-231 and Hs578T (*in vivo*)	Size: ∼174 nm; zeta: +25 mV; and RNA EE: confirmed	∼1000× miR-200c upregulation; significant inhibition of migration/invasion; and no systemic toxicity	Transient miR effect, variable response by cell line, small animal n, and no long-term toxicity	2021 (ref. 114)
eEV-PNC (uPA-targeted antimiR-21/-10b PLGA NPs)	4T1 (*in vivo*) and BALB/c mice	Size: ∼250 nm; EE: 70%; and zeta: not specified	2.8× More antiproliferative *vs.* DOX; 40% complete tumor regression; survival ↑; and no systemic toxicity	Off-target accumulation, complex prep, no synchronized release, and partial tumor-specificity	2022 (ref. 113)
C-PEG-Nio-AuNP/miR-33a	MCF-7	Size: ∼112–118 nm; zeta: +49–56 mV; and AuNP EE: 82.41%	73% Apoptosis, high Bax/Bcl2 ratio, and good 90 days stability	*In vitro* only, no normal cell data, relies on the EPR effect, and no targeting ligands	2024 (ref. 109)
AuNP/miR-204-5p (free NPs)	MCF-7	Size: 28.3 nm; zeta: −32.2 mV; and PDI: 0.435	Non-toxic ≤0.6 µg mL^−1^; MMP-9 ↓, NF-κB ↓ *via* miR-204-5p; and anti-inflammatory effect	Only MCF-7, no *in vivo*, short-term (72 h), and no pharmacokinetics or off-target data	2023 (ref. 110)
PEG-AuNP/miR-206	MCF-7	Size: not specified; zeta: +12.5 mV; and miR binding ∼76%	∼80% Viability reduction at 48 h; apoptosis induction; and G0/G1 arrest	No *in vivo*, short-term only, no long-term efficacy, unclear systemic toxicity, and incomplete caspase data	2022 (ref. 111)
MSN-PEI-miR-200c-HA	MDA-MB-231 (*in vitro* and *in vivo*)	Size: not specified (aggregated to ∼400 nm in serum); and miRNA loading: 0.7 wt%	EMT inhibition, reduced invasion, G2 arrest, tumor shrinkage, and no *in vivo* toxicity	Protein corona, off-target organ distribution, low miR content, PEI toxicity risk, and *in vivo* stability	2023 (ref. 112)

#### 2.1.2. Ovarian and cervical cancer

Engineered EV-mimetics comprise minimal components to replicate the essential characteristics of natural EVs for sustained circulation. These designed synthetic EVs are proficient at loading and delivering medicines to specific target cells. The inaugural clinically utilized liposome-based product employing 1,2-dioleoyl-*sn*-glycerol-3-phosphatidylcholine (DOPC) for the treatment of malignant meningitis, Depocyt®, received approval in 1999.^117^ Paclitaxel and doxil, which were authorized to treat recurrent ovarian cancer in 1999, are two of the numerous liposomal formulations that have been clinically authorized for ovarian cancer chemotherapy.^118–120^

**Fig. 8:**
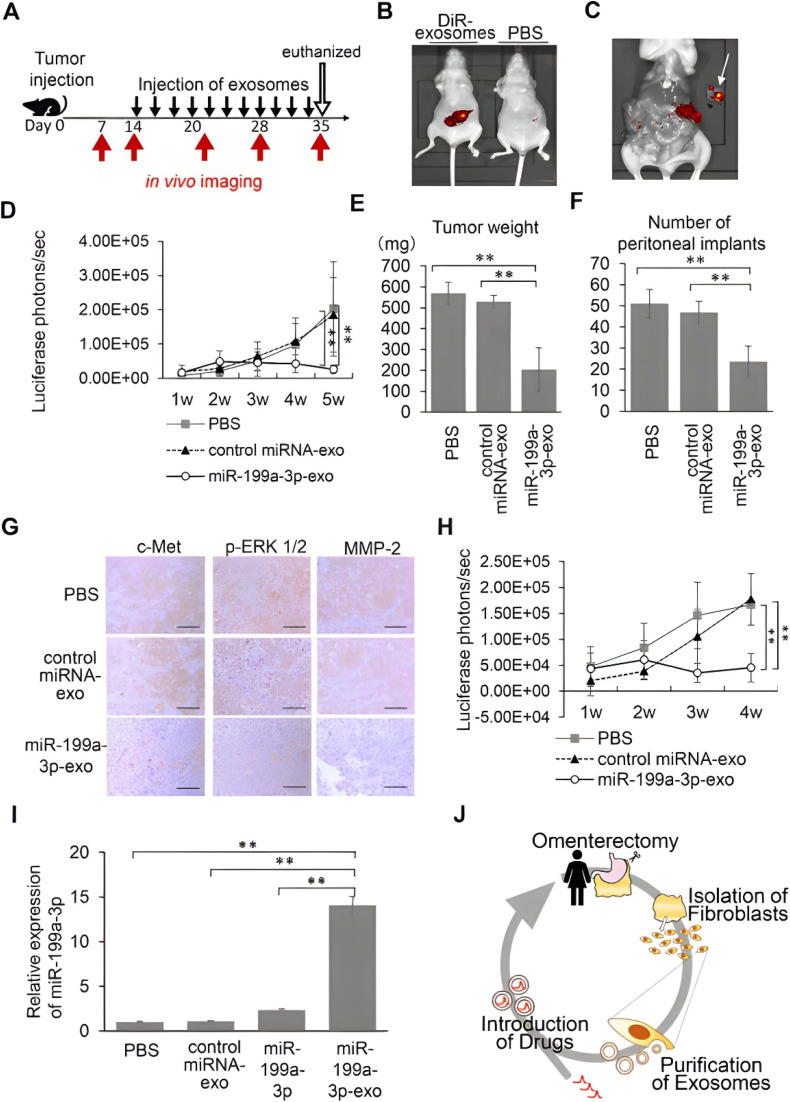
Efficacy of the miR-199a-3p-loaded exosomes in xenograft ovarian cancer model mice. (A) Schematic showing the schedule of the *in vivo* experiments. (B) Representative pictures taken by the IVIS imaging system of tumor-inoculated animals (left, DIR-labeled exosomes and right, PBS) at 5 weeks after inoculation with SKOV3-13 cells. (C) Enlarged picture of the mouse in (B) after laparotomy. The arrow indicates the resected tumor. (D) Luciferase activities of peritoneal tumors of mice injected i.p. with PBS, control miRNA-loaded exosomes, or miR-199a-3p-loaded exosomes. Five weeks after inoculation, the mice were sacrificed and (E) their tumor total burden and (F) number of peritoneal implants were confirmed. These results are expressed as mean ± SD; PBS group, *n* ¼ 10; control miRNA-loaded exosome group, *n* ¼ 9; and miR-199a-3p-loaded exosome group, *n* ¼ 8. (G) Immunohistochemical analysis of the peritoneal implants. Scale bar = 200 mm. (H) Luciferase activities of the peritoneal tumors of mice injected intravenously with PBS, control miRNA-loaded exosomes or miR-199a-3p-loaded exosomes measured weekly. The results are expressed as mean ± SD; PBS group, *n* ¼ 6; control miRNA-loaded exosome group, *n* ¼ 5; and miR-199a-3p-loaded exosome group, *n* ¼ 5. (I) miRNA qRT-PCR with mouse plasma. PBS, control miRNA-loaded exosomes, miR-199a-3p, or miR-199a-3p-loaded exosomes were injected intravenously into female athymic BALB/c nude mice. Fifteen minutes after the injection, miR-199a-3p levels in plasma were determined using qRT-PCR. (J) Suggested treatment model using exosomes from fibroblasts derived from patients' omenta. (Note: the correct spelling of the word in part (J) should be Omentectomy, which is spelled wrongly in the original figure.) **, *P* < 0.01. Reprinted with permission from ref. 121. Copyright, 2025. Published by Elsevier.

Kobayashi *et al.* purified exosomes from ovarian cancer patient-derived omental fibroblasts and loaded them with miR-199a-3p; Alexa Fluor 488-conjugated miR-199a-3p was synthesized. Primary human omentum-derived fibroblasts were used as a source of endogenous exosomes (around 100 nm), which expressed classical vesicular markers (CD9, CD63, and CD81) and lacked intrinsic tumor-promoting effects. Purified exosomes were loaded with miR-199a-3p, a tumor-suppressive miRNA, by electroporation, which allowed for the stable encapsulation of miRNA without altering the vesicle morphology. Fluorescence tracking showed that loaded exosomes were efficiently taken up by ovarian cancer cells, whereas free miRNA alone did not enter the cells. Exosome-mediated delivery of miR-199a-3p resulted in a significant increase in intracellular miRNA levels (up to around 67 000-fold), which shows the high efficiency of delivery. This system suppressed cancer cell proliferation and invasion by reducing mesenchymal–epithelial transition factor (c-Met) expression and inhibiting hepatocyte growth factor (HGF)-induced ERK and Akt phosphorylation. miR-199a-3p expression increased 8592-fold in CaOV3, 67 188-fold in SKOV3ip1, and 2280-fold in the OCVCAR3 cell lines of the ovary. The optimal dose was 100 µg mL^−1^. In *in vivo* models, intraperitoneal or intravenous administration of miR-199a-3p-containing exosomes resulted in preferential tumor accumulation, increased circulating miRNA stability, reduced peritoneal tumor burden, and inhibition of the c-Met signaling pathway in xenografts, demonstrating the efficacy of this nanocarrier for therapeutic miRNA delivery. Mice treated with miR-199a-3p-Exo had significantly lower tumor weights (203.3 ± 106.0 mg) after intraperitoneal injections every 48 hours than the phosphate-buffered saline (PBS) (569.8 ± 53.5 mg) and control miRNA-Exo (529.6 ± 30.8 mg) groups. Furthermore, compared to the PBS and control groups, the miR-199a-3p-Exo group had significantly fewer peritoneal implants (23.6 ± 7.4) (Fig. 8). Despite hopeful xenograft model results, more preclinical safety and pharmacokinetic data are needed before clinical use. The findings are restricted to ovarian cancer models, and their relevance to other cancers remains untested. Additionally, scalability, cost, or regulatory challenges are not discussed for producing exosome-based therapies.^121^

A dextran-based lipid nanogel (LNG) system was synthesized using a graft copolymerization-induced self-assembly (GISA) approach. LNGs were fabricated *via* GISA, where hydrophobic poly(methyl acrylate) chains induced self-assembly of the nanogel and disulfide linkage stabilized the structure in a reducing environment-sensitive manner. Cationic amphiphilic lipids were then assembled on the surface of the nanogels, creating a hybrid nanocarrier that could simultaneously load paclitaxel and siRNA. Paclitaxel was encapsulated in the nanogel core through hydrophobic interactions, while siRNA was electrostatically loaded into the lipid shell. After endocytosis, the cytoplasmic reducing environment caused the nanogel to degrade and siRNA to be released; siRNA reduced drug resistance by silencing multidrug resistance protein 1 (MDR1) and enhanced the efficacy of paclitaxel. LNGs accumulated in the tumor in *in vivo* models through the EPR effect, providing a more effective combination therapy. The siRNA encapsulation efficiency was 89.6% ± 3.7%. LNGs exhibited high cellular uptake in both OVCAR3 and drug-resistant DROV cells, and MDR1 gene knockdown was efficient using siRNA1, especially in drug-resistant DROV cells. Combination therapy with LNGs-siRNA and paclitaxel reduced cell viability more than single-agent treatments. No significant body weight loss or organ damage, indicating good biosafety. They reported that transmission electron microscopy (TEM) showed incomplete membrane structures in paclitaxel-loaded LNGs, indicating that hydrophobic paclitaxel weakened the lipid shell, which can affect stability. LNGs mostly stay in the cytoplasm due to their size, which can limit access to nuclear targets. Additionally, the drug loading percentage was 1.35%, which may limit the therapeutic payload per carrier.^122^ In another study, Pisano *et al.* used immune-derived exosome mimetics (IDEM) loaded with DOX to examine a scalable delivery strategy against ovarian cancer. A prolonged drug release profile and high drug entrapment efficiency were demonstrated by the EV-mimetics. In addition, DOX-loaded IDEM outperformed the free anticancer agent in terms of *in vitro* cytotoxicity and apoptotic effect.^123^

A nanostructure was designed using DNA tubular origami (DTO) and fluorescence dramatically increased after exposure to miR-21, indicating DTO opening. The DTO nanocarrier was designed as a 3D tubular structure of DNA origami and stabilized in the closed state by capping strands at both ends. This architecture prevented unwanted kinking and edge misalignment and allowed for the controlled incorporation of 20 internal binding sites for cargo or fluorescent labels. DTO was synthesized by using a one-pot method based on complementary base pairing. The nanostructure underwent structural unfolding in the presence of miR-21, which was overexpressed in ovarian cancer cells, and showed a significant increase in fluorescence signal. The approximately 120 nm length of the DTO made it suitable for tumor accumulation *via* the EPR effect. Cellular uptake and live imaging studies showed that DTO was selectively internalized in cells overexpressing miR-21, and this process was likely related to caveolin-1-dependent endocytosis. Flow cytometry was used to analyze 15 000 cells, and the ovary's SKOV3/DDP and A2780/DDP cell lines demonstrated the highest levels of miR-21 expression and effective DTO internalization. 2780/DDP demonstrated quick uptake within 0.5 hours, while SKOV3/DDP increased gradually over 3 hours. Normal ovarian epithelial cells (IOSE80) were hardly absorbed. Despite some structural differences and nonspecific membrane binding in IOSE80, the DTO was able to effectively target cancer cells *in vitro*. Obstacles include dimerization during unzipping and the lack of *in vivo* testing.^124^

Salamone *et al.* created pristine AuNPs and reported that the successful non-covalent loading of trastuzumab (TZ) onto hydrophilic AuNPs resulted in stable nanoconjugates with a 41% ± 4% loading efficiency. The AuNPs-TZ displayed a time-dependent and miR-200c-specific sensitization effect by reducing viability in miR-200c-transfected SKOV3 cells, especially at 0.1 µg mL^−1^ after 48 hours, but not cytotoxicity alone. Apoptosis tests showed that miR-200c-transfected cells treated with AuNPs-TZ had a synergistic increase of 25.2% in late-stage apoptosis. Moreover, the HER-2/MAPK signaling pathway was markedly downregulated, and SKOV3 cells developed epithelial differentiation characteristics as a result of the combination of AuNPs-TZ and miR-200c.^125^ Additionally, flower-shaped silicon dioxide–polyethylenimine (SiO_2_–PEI) NPs were synthesized using a high plasmid DNA (pDNA) and siRNA loading capacity in order to deliver the tumor suppressor miR-let-7c-5p to HeLa cells. With little cytotoxicity, this method efficiently transferred miR-let-7c-5p, preventing the spread of cancer. According to their findings, a promising anticancer strategy for cervical cancer was represented by flower-shaped SiO_2_-PEI NPs loaded with let-7c-5p (FSP-let-7c-5p NPs), which targeted the β-catenin/SLUG and miR-let-7c-5p/insulin-like growth factor 1 receptor (IGF-1R)/PI3K/AKT pathways.^126^

Table 3 summarizes the NP-based miRNA delivery systems used in ovarian and cervical cancer research studies.

**Table 3:** Summary of the nanoparticle-based miRNA delivery systems used in ovarian and cervical cancer research studies

Aspect	Dextran-based lipid nanogel	Fibroblast-derived exosomes	DNA tubular origami-DTO
Nanocarrier type	Dextran-based lipid nanogel (LNG) synthesized *via* GISA	Exosomes isolated from omental fibroblasts	DNA tubular origami (DTO) constructed with 104 staple strands
Particle size	∼100 nm (TEM-confirmed lipid bilayer)	∼100 nm (electron microscopy and nanoparticle tracking)	Length = ∼120 nm and width = 13–18 nm (closed) and ∼60 nm (opened/dimerized)
Drug/siRNA loading	PTX drug loading: 1.35%; EE 93%; and siRNA EE 89.6% ± 3.7%	Electroporated miR-199a-3p into exosomes	20 miR-21 binding sites with FAM-labeled handles
Cell uptake	High uptake in OVCAR-3 and drug-resistant DROV cells and enhanced in DROV *via* P-gp efflux bypass	No tumor-promoting effect of fibroblast exosomes on OC cells	Rapid uptake in A2780/DDP cells; slower but stronger over time in SKOV3/DDP; and minimal in IOSE80
Gene silencing/effect	Efficient MDR1 knockdown in DROV; lowered PTX IC50; and combo therapy improved viability reduction	miR-199a-3p suppressed OC proliferation, invasion; downregulated c-Met, mTOR, IKKβ, and CD44	miR-21-triggered DTO unzipping and fluorescence increase and DTO internalized and opened in cancer cells
*In vivo* efficacy	LNGs-PTX-siRNA reduced tumor volume; no body weight loss; and confirmed apoptosis (H&E, TUNEL, PCNA, Ki67)	miR-199a-3p-Exo reduced tumor weight (203.3 ± 106.0 mg *vs.* controls >500 mg), fewer peritoneal implants; and stable plasma levels	No *in vivo* data reported
Key quantitative data	siRNA EE 89.6% ± 3.7%; PTX DL 1.35%; and tumor weight reduction significant	miR-199a-3p fold increase: CaOV3 (8592×), SKOV3ip1 (67 188×), OVCAR3 (2280×); and tumor weight ↓ ∼64%	DTO length ∼120 nm; fluorescence measured every 10 s × 100; and 15 000 events per sample flow cytometry
Challenges/limitations	Membrane integrity disruption by PTX; moderate drug loading; omitted some *in vivo* controls; knockdown varies with siRNA sequence; and limited nuclear entry	Electroporation may damage exosome membranes; limited long-term and safety data; systemic biodistribution not fully explored; and scalability issues	Structural deviations due to surface effects; dimerization during unzipping; imaging overlap (DAPI/Cy3); no *in vivo* data; uptake varies by cell line; and nonspecific binding in normal cells
Ref.	122	121	124

### 2.2. microRNA-based nanotechnology in reproductive disorder treatment

#### 2.2.1. Endometriosis

Egorova *et al.* used RNA interference (RNAi) therapy as an anti-angiogenic endometriosis treatment. The Arg-Gly-Asp peptide 1 conjugated with oligoarginine R6 (RGD1-R6) peptide nanocarrier formed stable nanoscale complexes that protected the siRNA from enzymatic degradation by utilizing electrostatic interactions between the siRNA and the R6 peptide. The cross-linking of R6 reduced the particle size and improved the physicochemical properties. The presence of the iRGD ligand enabled the recognition and specific binding to the αvβ3 integrin, facilitating the targeted entry of the complexes into endothelial and cancer cells expressing this receptor. After cellular internalization, the anti-vascular endothelial growth factor A (VEGFA) siRNA caused a specific reduction in VEGFA mRNA expression and protein secretion, which ultimately led to the inhibition of endothelial cell migration and suppression of angiogenesis. These effects were also confirmed in an animal model of endometriosis by reducing angiogenesis and lesion volume. In all tested cell lines, the RGD1-R6/siRNA complexes demonstrated low cytotoxicity, with over 80% cell viability at N/P ratios of 8/1 and 16/1, and only slight toxicity was observed in the endothelial EA.hy926 cells at a higher 24/1 ratio. In tests of gene silencing, green fluorescent protein (GFP) expression dropped to 46.7% (8/1), 34.3% (16/1), and 50.9% (24/1) in MDA-MB-231 cells, while VEGFA mRNA levels in EA.hy926 cells dropped to 51.8% (8/1) and 42.8% (16/1). Protein analysis confirmed a significant decrease in VEGFA secretion with only RGD1-R6/anti-VEGFA complexes. As a result, wound closure dropped to 25.9% (8/1) and 22.6% (16/1). The treatment significantly reduced the volume of the endometriotic implant by 2 times (*P* < 0.01), reduced the expression of the VEGFA gene to 52.1% ± 4.5%, and reduced angiogenesis *in vivo*. However, limitations remain—slight cytotoxicity at high doses, reduced efficacy in serum-containing media, and potential non-specific effects due to excessive integrin binding. Additionally, results from subcutaneous rat models may not fully predict outcomes in human endometriosis.^127^

In a study by Liang *et al.*, the miR-200c mimic was used for *in vivo* delivery *via* a PEI–PEG–RGD nanocarrier. PEI, a cationic component, electrostatically complexed and stabilized the miRNA, allowing for systemic injection. PEG improved carrier performance by increasing biocompatibility and circulatory stability, while the RGD peptide induced preferential accumulation of the nanocarrier in endometriotic lesions, as confirmed by near-infrared imaging. Successful delivery of miR-200c *via* this nanocarrier resulted in a decrease in fluorescence signal intensity and a reduction in the volume of endometriotic cysts. They reported that restoring miR-200c levels in human endometrial stromal cells (HESCs) resulted in a significant decrease in cell migration and proliferation (*P* < 0.01) while inhibiting it had the opposite effect. miR-200c was specifically directed against the lncRNA metastasis-associated lung adenocarcinoma transcript 1 (MALAT1), which was significantly upregulated in ectopic tissues (*P* = 0.0477) and correlated negatively with miR-200c expression (*r* = −0.8176, *P* < 0.0001). Two distinct binding sites were validated by luciferase assays. miR-200c inhibition reversed the effects of MALAT1 knockdown or miR-200c overexpression, which suppressed epithelial–EMT by upregulating E-cadherin and downregulating ZEB1, ZEB2, and N-cadherin. Treatment with the miR-200c mimic dramatically decreased lesion volume and fluorescence intensity *in vivo* using a rat model of endometriosis, whereas the inhibitor had the opposite effect. Although the results are encouraging, they should be interpreted with caution because the study was limited by a small initial microarray sample, substantial reliance on *in vitro* assays that may not fully mimic *in vivo* conditions, and the use of a rat model that may not entirely recapitulate the biological behavior of human endometriosis.^26^

PLGA-based NPs were developed to deliver miRNA-503 to endometriotic cyst stromal cells. miRNAs 503 and primer were purchased from Pishgam Co. (St Kargar, Tehran, Iran). PLGA formed NPs with a size of less than 100 nm, which increased in size after complexation with miRNA and remained suitable for gene delivery. The addition of PEI increased the positive surface charge of the complexes, which enhanced the electrostatic interaction with the cell membrane and facilitated the entry of NPs into endometriotic stromal cells through endocytosis; this process was confirmed by TEM. After entering the cells, the PLGA/miRNA-503 complex caused a dose- and time-dependent decrease in cell viability; at 75 µM for 48 hours, cell viability was 89.9% *versus* 98.4% in the controls. Apoptosis rose significantly to 35.7% with PLGA/miRNA, compared to <1% in controls. *In vivo*, the treated lesions appeared smaller, with more apoptosis. Although effective, the system's limitations include dose-dependent cytotoxicity and a narrow therapeutic window, with long-term effects yet to be studied.^128^

#### 2.2.2. Placental disorders

Generally, lipid nanoparticles (LNPs) include ionizable lipid, helper phospholipid, cholesterol, and PEG-lipid; the main functions of ionizable lipids include the packaging of RNA therapeutics into LNPs as well as potentially facilitating their escape from the endosome; phospholipids, a type of helper lipid, play an important role in maintaining the structural stability of LNPs during storage and circulation in the body; PEG-containing lipids are mainly associated with preventing the aggregation of NPs and increasing their stability during formulation and storage.^129^ LNPs are a versatile drug delivery platform capable of packaging and carrying various types of therapeutic drugs, including small molecule drugs, proteins, and RNA-based drugs.^130^ As a new generation of drugs, mRNA-based drugs have significantly transformed the modern medical landscape, especially due to their rapid development and adoption during the COVID-19 pandemic (Pfizer/BioNTech and Moderna COVID-19 mRNA vaccines in 2020).^131^ Compared to conventional protein-based therapeutics, which require time-consuming steps of prior production and purification, mRNA allows the protein of interest to be expressed directly inside the cell based on its easily programmable nucleotide sequence.^132^ Patisiran (Onpattro®) was the first siRNA-based drug to receive Food and Drug Administration (FDA) approval in 2018 using LNPs, practically demonstrating the clinical efficacy of RNA delivery *via* LNPs in the treatment of hereditary transthyretin amyloidosis.^131,133^ Recently, Li *et al.* reported that LNPs can deliver mRNA (and to a limited extent self-amplifying RNA [saRNA]) to the retina, but protein expression remains low, while the ionizable lipid composition plays a decisive role in inducing a local immune response, highlighting the need to simultaneously optimize delivery efficiency and safety.^134^ Additionally, in the study, topical delivery of miR-29b using LNPs significantly reduced corneal fibrosis, with the expression of fibrotic markers, including α-smooth muscle actin (α-SMA) and collagens, inhibited, and the arrangement of stromal collagen fibers improved. In addition, LNP-miR-29b facilitated corneal structural remodeling and improved wound healing in animal models by accelerating epithelial repair.^135^ Another recent study showed that intra-articular delivery of lncRNA growth arrest-specific 5 (GAS5) using LNPs significantly improved the repair of osteoporosis-related rotator cuff tears in animal models and promoted mechanical function and tendon-bone morphology by improving bone quality and inducing macrophage polarity to the M2 phenotype.^136^

In the field of women's health, placental dysfunction underlies many pregnancy-related diseases, which has led to increased attention to the development of targeted RNA delivery strategies to the placenta using LNPs. Recently, several studies investigated the use of LNP formulations for targeted mRNA delivery to the placenta; for example, in the study by Safford *et al.*, LNP C5 exhibited a 4-fold increase in mRNA delivery efficiency compared to a standard formulation and *in vivo* intravenous administration of LNP C5 to pregnant mice improved mRNA delivery to the placenta.^131^ Moreover, Swingle *et al.* identified LNP 55, which performed more than 100 times better than the FDA-approved Onpattro LNP in terms of mRNA delivery to the placenta in pregnant mice. It was believed that LNP 55's targeted delivery mechanism, which enabled selective delivery to the placenta, involved β2-glycoprotein I adsorption. In preeclampsia models caused by inflammation and hypoxia, LNP 55 encapsulating VEGF mRNA improved fetal health, placental vasculature, and immune function while lowering maternal hypertension.^137^

Nanocarriers have potential beyond relying solely on the EPR effect in reproductive disorders; for example, in endometriosis, RGD-based ligand-tagged carriers targeting αvβ3 enabled selective accumulation and efficient entry of si/miRNA into endothelium and stromal cells, and PEI- or PLGA-based polymeric carriers enhanced gene knockdown efficiency and apoptosis induction by enhancing endocytosis.^26,127,128^ Moreover, in the placenta, some LNPs showed differential placental distribution and improved fetal and maternal indices by binding to proteins such as β2-glycoprotein I. These results underscore the practical advantage of molecular targeting and relying on physiological properties of the target tissue although dose-dependent toxicity, heterogeneity of receptor expression, and limited generalizability of animal models still hinder clinical translation.^131,137^

## 3. Challenges in microRNA delivery systems for cancer and reproductive health therapies

Despite the attention surrounding miRNAs and their role in disease development or health maintenance in the human body, their efficacy has been limited due to their poor targeting ability, short circulation time in the body, and off-target effects in free miRNA-based agents. To overcome these challenges, miRNA-carrying NPs have been proposed as a promising strategy, as NPs protect the loaded agent from the external environment, which prevents its inactivation or degradation, thereby increasing circulation time in the body and improving targeted accumulation in the target tissue.

Polymer NPs have been widely investigated for miRNA delivery due to their structural diversity and the possibility of fine-tuning their physicochemical properties. Cationic polymers such as PEI, by virtue of their strong electrostatic complexation and the “proton sponge” effect, have provided effective endosomal escape and have resulted in tumor growth suppression in preclinical models through the delivery of tumor suppressor miRNAs; however, their high positive charge density, lack of biodegradability, and dose-dependent toxicity have limited their clinical application.^138^ In contrast, biodegradable polyesters such as PLGA and poly(ε-caprolactone) (PCL) have offered higher biosafety and have enabled controlled release, but due to their negative surface charge, they often require cationic modifications or hybrid systems for efficient miRNA loading.^113,139,140^ LNPs have shown an intrinsic ability to interact with the cell membrane and facilitate miRNA entry into the cell due to their structural similarity to the cell membrane. In these systems, spontaneous electrostatic complexation between cationic lipids and miRNA resulted in effective aggregation and protection against enzymatic degradation. Furthermore, the use of helper lipids such as cholesterol and 1,2-dioleoyl-*sn*-glycero-3-phosphoethanolamine (DOPE), as well as PEGylation, has significantly improved the stability, circulation time, and biosafety of LNPs.^26,141^

siRNA-based therapeutic approaches induce an RNAi response by introducing synthetic siRNA strands into target cells, thereby inhibiting the expression of a specific mRNA to achieve a gene silencing effect. In contrast, miRNA-based therapies include two main strategies: miRNA inhibition and miRNA replacement. In the inhibition approach, a similar function is pursued as antisense therapies because synthetic single-stranded RNAs act as miRNA antagonists (also known as antagomirs or anti-miRs) by inhibiting endogenous miRNAs and neutralizing their effect. In the replacement approach, synthetic miRNAs (or miRNA mimics) are used to mimic the function of naturally occurring endogenous miRNAs.^142^ One of the fundamental differences between siRNA and miRNA is that siRNA usually recognizes only a specific target site on a single mRNA, thereby inhibiting the expression of a specific gene. In contrast, miRNAs, because their mRNA recognition depends on binding to a short and limited segment of the RNA sequence, can simultaneously target multiple different mRNAs and regulate the expression of multiple genes. To initiate the RNAi process, siRNA must have complete complementarity with its target mRNA, while miRNAs can bind to multiple mRNAs with incomplete complementarity. In addition, the mechanism of action of the two is also different; siRNA acts by directly cleaving and degrading mRNA, while miRNA mainly reduces gene expression by inhibiting the translation process.^143^ One of the inherent challenges of miRNA-based therapies is the occurrence of off-target effects and their associated toxicities, which arise from the ability of each miRNA to simultaneously regulate the expression of different genes and are recognized as one of the most important obstacles to the development of miRNA-based therapies. Therefore, further research is necessary to optimize the design and application of miRNAs and develop them as effective strategies in cancer treatment.^144^ Furthermore, the effective dose of miRNAs, control of tissue distribution, and their long-term safety monitoring remain more challenging than siRNA/mRNA.

Numerous important obstacles limit the therapeutic potential of miRNA delivery, especially when combined with NPs in cancer and reproductive health treatments. One of the limitations of utilizing nano-based delivery systems is their stability. However, the hydrogel component stabilized MnO_2_@Ce6 in the study by Wang *et al.*^116^ This system could still face stability challenges in complex biological environments. Additionally, significant degradation of biological activity for NL-miR-1296 was reported after 80 days at common storage temperatures (25 °C and 4 °C) by Albakr *et al.*^115^ Moreover, the uniformity of the created NPs remains an important factor in their reproducibility and scalability. The moderate polydispersity (PDI = 0.435) of the system designed by Farhana *et al.*^110^ implies that the NP size distribution was not highly uniform, which could have affected the outcomes. Moreover, most of the systems had a complex multi-step production process, including drug loading, miRNA conjunction or aptamer modifications,^116^ which could present scalability challenges for clinical translation. Rapid degradation and clearance of miRNAs in the bloodstream, limited penetration through biological barriers, such as the tumor microenvironment and blood–brain barrier, and endosomal entrapment limit functional release. The poor uptake of free miRNA in experiments, as reported by Schilb *et al.*,^114^ together with the rapid decline of miR-200c levels after 4 days and the significant drop by day 14, highlights the instability of miRNA in biological systems and the necessity for repeated dosing. Notably, clinical translation is made more difficult by pharmacokinetic restrictions and the difficulty of scalable, reproducible manufacturing.

Loading miRNAs into EVs, which are frequently employed for tumor-targeting applications, presents another significant challenge. Although the functionality of EVs has improved due to bioengineering advancements, non-specific interactions can lead to less precise targeting and undesired accumulation in non-tumor tissues, and loading efficiency is still limited. Additionally, when NPs are exposed to biological fluids, a protein corona is formed around them, which makes it more difficult for them to target and affects their capacity to deliver miRNAs to cancer cells specifically.^112,113^ Furthermore, engineered EV-mimetic NPs, which mimic the properties of natural EVs, face hurdles such as rapid clearance by macrophages, reducing their therapeutic potential. The need for optimization is highlighted by this immune recognition and the corresponding low therapeutic efficacy. Furthermore, it is difficult to increase the production of these NPs while preserving their high loading efficiency and homogeneity.^145^

AntimiRNA accumulation in non-tumor organs (liver, spleen, and kidneys) in the study by Bose *et al.*^113^ suggested incomplete tumor specificity. These challenges can limit the potential for dose escalation in aggressive tumors. The utilization of structures like CpG sequences may induce immune responses, but not all studies have assessed immune-related outcomes, specifically in the long term.^110,114,116^ Additionally, most of the utilized cell lines in recent studies included MDA-MB-231, Hs578T, OCVCAR3, or SKOV3,^114,115,121,124,127^ in which each study focused on one or two lines in a single cancer type. There is a need for more evaluations of the usage of the developed systems in other cell lines and cancer types for broader applicability.

Most studies had a limited duration of follow-up, varying from 48 hours to 90 days,^109,111,114,121,128^ which leads to limited data about the long-term toxicity, off-target effects, and biosafety that limit the potential of clinical utility. Moreover, the present experiments are limited to *in vitro* trials, and for more precise data, there is a need for more clinical experiments. In addition, a small animal sample size (standard in preclinical work) leads to potential limitations for statistical robustness.^146^

For future studies, it is necessary to explore strategies to enhance the stability and reproducibility of miRNA-based NPs, accompanied by addressing the limited targeting precision through tissue-specific ligands or advanced NP formulations and insufficient drug delivery. Additionally, novel strategies to increase the miRNA loading efficiency of EVs may enhance their clinical applicability. Furthermore, to gain a better understanding of their safety profiles, long-term studies assessing immune responses are crucial.

Biological research has changed as a result of artificial intelligence's (AI) ability to efficiently glean insights from complex data. Machine learning (ML) techniques, particularly deep learning approaches, can be used to identify critical miRNAs across various cancers and create prognostic models. Additionally, AI integration has led to the creation of comprehensive miRNA databases for identifying mRNA and gene targets, facilitating a better understanding and application in cancer research. These methods significantly enhance the identification of diagnostic and prognostic biomarkers by enabling the precise prediction of miRNA targets, subcellular localization, and miRNA-disease associations.^147^ Furthermore, by identifying connections between miRNAs, medications, and illnesses, ML reduces experimental costs and speeds up drug discovery, all of which contribute to the development of miRNA-targeting therapies.^148^ Additionally, AI facilitates integrative analyses of multi-omics data, which advances our knowledge of miRNA biology and its potential applications in therapy. These developments are gradually changing the field of miRNA research and are anticipated to have a greater influence on personalized medicine and clinical diagnostics for cancer and other illnesses.^149^

## 4. Conclusion

miRNAs are not only disease markers but also key regulators of women's health across different life stages. Due to the critical role of miRNAs in the regulation of reproductive processes, any dysregulation in the expression level of miRNAs leads to disorders such as endometriosis, PCOS, pregnancy outcomes, and malignant changes in the reproductive system. Designing nanostructures like PEGylated nanohydrogels, PLGA-PEG NPs, PEG nanogels, MSNs, AuNPs, EV mimetics, SiO_2_-PEI NPs, DTO, and liposomes for delivering miRNAs to target tissues for inhibiting the progression of diseases offered more precise and effective treatments while minimizing side effects for treating ovarian, cervical, and breast cancers, as well as endometriosis and placental disorders. However, the advancements in miRNA-based interventions have been accompanied by challenges such as stability, delivery efficiency, and off-target effects, necessitating advanced strategies.

## Author contributions

Neda Farzizadeh: conceptualization, investigation, writing – review and editing, and visualization; Zahra Najmi: conceptualization, supervision, and writing – review and editing; Morteza Amoozgar: funding acquisition; Mona Aminbidokhti: supervision and writing – review and editing; Amirali Hariri: supervision and writing – review and editing; Arezoo Khosravi: visualization and writing – review and editing; Siavash Iravani: conceptualization, writing – review and editing, visualization, and supervision; and Ali Zarrabi: conceptualization, writing – review and editing, visualization, and supervision.

## Conflicts of interest

The author(s) declare no conflicts of interest.

## Data Availability

No primary research results, software or code have been included, and no new data were generated or analyzed as part of this review.
